# Taxonomic review of Argentine mackerel* Scomber japonicus* (Houttuyn, 1782) by phylogenetic analysis

**DOI:** 10.22099/mbrc.2017.25981.1276

**Published:** 2017-09

**Authors:** María Inés Trucco, Claudio César Buratti

**Affiliations:** Instituto Nacional de Investigación y Desarrollo Pesquero (INIDEP) Paseo Victoria Ocampo Nº1, Escollera Norte, B7602HSA Mar del Plata, Provincia de Buenos Aires, República Argentina

**Keywords:** Phylogeny, COI, *Scomber japonicas*, Argentina

## Abstract

Taxonomically, Argentine mackerels were first considered as *Scomber japonicus marplatensis* and later as *Scomber japonicus* Houttuyn 1782, although, in the last years, different studies have suggested that South Atlantic mackerel species belongs to *Scomber colias* Gmelin 1789. These latter results, incorporated in the main fish databases (FishBase and Catalog of Fishes), promoted a phylogenetic study using cytochrome c oxidase I (COI) gene sequences taken from the Barcode of Life (FISH-BOL) database. Thus, 76 sequences of *S. japonicus*, *S. colias*, *S. australasicus* and *S. scombrus* from different regions were used; including 3 from *Sarda sarda* as outgroup. Among *S. japonicus* selected sequences are those corresponding to the Argentine mackerels collected in 2007. Phylogenetic trees were obtained by neighbor joining and maximum likelihood methods and a network of haplotypes was reconstructed to analyze the relationship between species. The results showed the clear differentiation of *S. australasicus*, *S. scombrus* and *S. japonicus* from the Pacific while *S. japonicus* from Argentina was included in the *S. colias* group, with genetic differences corresponding to conspecific populations (0.1%). Four of the five Argentine specimens shared the same haplotype with *S. colias*, and none were shared with *S. japonicus* from the Pacific. These results suggest that the current specific name of Argentine mackerel *S. japonicus* should be changed to *S. colias*, in agreement with several genetic studies carried out with species of the genus *Scomber*.

## INTRODUCTION

The Scombridae family includes species of great commercial interest as tuna, bonitos and mackerels [1]. The taxonomy of this family has been revised many times and, even today, there is no consensus as to its conformation. Recently, one of the most studied genera is *Scomber*, which usually is described as composed by three species: *S. scombrus*, *S. japonicus* and *S. australasicus*. Of these, *S. scombrus* is the most differentiated since it lacks a swim bladder, has more ossified bones and the first hemal spine is situated before the first interhemal bone [2]. On the other hand, due to the wide distribution of *S. japonicus* in Atlantic, Indian and Pacific oceans, new species or subspecies has been identified [2-6]. In this context, *S. colias* was described from Atlantic waters, first, as a synonym for *S. japonicus* [1] and, more recently, there is strong support for the genetic distinctiveness of *S. japonicus* and *S. colias* [7-9]. The complex taxonomic classification of the genus *Scomber* has been reflected too in some fish databases: FishBase [10] and the Catalog of Fishes [11] cited, as belonging to the genus *Scomber*, 131 and 47 species, respectively, both agreeing that, currently, there are only 4 valid species.

Species identification by genetic markers has clarified several aspects as classification, evolutionary origin and spatial distribution in the family Scombridae [12, 13]. Molecular markers have showed that *S. japonicus* is present in the Southern and Eastern Pacific, with differences between them [14, 15] and in the Mediterranean and Argentine sea, as well [16]. Later, it was established that *S. japonicus* from the Atlantic and Indo-Pacific were distinct species [17] and phylogenetic analyses showed that *S. colias* an *S. japonicus* were different species, one belonging to the Atlantic and the other to the Pacific [8, 9, 18,19]. 

 In Argentina, considering the presence of gas bladder, López [4, 20] resolved that mackerels should have a subspecies rank and named it *Pneumatophorus japonicus marplatensis*. Angelescu and Gneri [21] indicated that mackerels from Argentine Sea belonged to the genus *Scomber* and subsequently, Perrotta et al. [22] suggested that the number of individuals studied to expand the taxonomic rank to subspecies was sparse; hence the name *marplatensis *would not be right. This was corroborated years later in a study with enzymatic markers in *S. japonicus* from the Mediterranean and the Argentine Sea [16]. Since then, the Argentine mackerel has a species rank as *S. japonicus* Houttuyn, 1782. In FishBase, *S. japonicus* appears as native of Argentina, but with the caveat that this is a wrong record, as well as in Uruguay and Brazil, being classified as *S. colias*.

Mackerel in Argentina has local consumption by canned production as its main destination in the city of Mar del Plata. In smaller volumes, it has been exported as whole frozen fish, mainly to Brazil, Nigeria, Ghana and Ivory Coast, among others [23]. Given the economic importance of this resource, it is necessary to clarify its taxonomic position, not only because the studies mentioned previously consider that South Atlantic mackerels should be named *S. colias* instead of *S. japonicus*, but also because it is an exported fish product on which genetic markers can be applied for traceability to prevent illegal fishing and fraud. Therefore, by using the COI mitochondrial gene, a phylogenetic analysis was performed with *Scomber* sequences from different geographical regions available in the Barcode of Life Data System (FISH-BOL). The reconstruction of a phylogenetic tree would allow analyzing the relationship between the sequences belonging to mackerels caught in the Argentine Sea and those corresponding to *S. japonicus* and *S. colias* obtained in other regions of the world.

## MATERIALS AND METHODS

Samples: Mackerel sequences from Argentina and those belonging to *S. japonicus*, *S. colias*, *S. australasicus* and *S. scombrus* were obtained from FISH-BOL database. As outgroup, *Sarda sarda* (bonito) sequences were downloaded from the same database. Geographic origin and accession number of species are indicated in Table 1. 

Phylogenetic analysis: Sequences (n= 76) were edited by BIOEDIT v.5.0.6 [24] and then a multiple alignment by CLUSTAL W was performed with the same program, yielding a sequence of 663 base pairs. Divergence values between COI sequences and the search for the phylogenetic tree were calculated by obtaining the evolutionary model that best fitted the data [25]. From the likelihood analysis obtained with MEGA v.6.0 [26] it was established that, following the Bayesian Information Criterion (BIC), the model that best agreed the data was Kimura-2 parameters distance model (K2-P) [27]. All positions containing gaps and missing values were eliminated. The Neighbor Joining tree (NJ), with distances (D) within and between populations through the K2-P model, was obtained with the same program, as well as the nodes robustness using a nonparametric bootstrapping method with 1000 replications [28]. A second phylogenetic tree was obtained by the General Time Reversible evolutionary model (GTR + G), chosen according to the corrected Akaike Information Criterion (AIC). 

Variability in DNA sequences was quantified by the segregating sites ratio, the haplotype diversity index (h) and nucleotide diversity index (π), using MEGA v.6.0 and DnaSP v.5.1 [29]; the latter was also used to identify haplotypes. A network was reconstructed for haplotypes with the median joining algorithm [30] by using Network 4.0 program, where each circle generated indicated a haplotype and its area was proportional to its absolute frequency.

**Table 1:** Species name, accession number, date and geographic origin of *Scomber* species and *Sarda sarda* sequences obtained from *FISH-BOL *database

**Species name**	**Sequence**	**Geographic origin**	**Lat. Long.**		**Year**
***Scomber japonicus***	FARG 481-08	CCOB-06-07- Argentina	-40.333	-60.433	2007
	FARG 482-08	CCOB-06-07- Argentina	-40.333	-60.433	2007
	FARG 486-08	CCOB-06-07- Argentina	-40.333	-60.433	2007
	FARG 487-08	CCOB-06-07- Argentina	-40.333	-60.433	2007
	FARG 495-08	CCOB-06-07- Argentina	-40.333	-60.433	2007
	BIM 145-13	Israel, Mediterranean Sea	32.877	34.96	2012
	BIM 146-13	Israel, Mediterranean Sea	31.738	34.543	2012
	BIM 179-13	Israel, Meditrerranean Sea	31.745	34.569	2012
	DNATR1340-13	Turquia, Marmara Sea	-	-	-
	DNATR1341-13	Turquia, Marmara Sea	-	-	-
	DNATR1342-13	Turquia, Marmara Sea	-	-	-
	ANGBF5365-12	China, South China Sea	17.311	113.276	2009
	ANGBF5366-12	China, South China Sea	23.125	117.876	2004
	ANGBF5367-12	China, South China Sea	21.125	113.42	2011
	ANGBF7066-12	SouthAfrica	-	-	2009
	ANGBF7155-12	SouthAfrica	-	-	2010
	ANGBF9523-12	Spain, Cantabric Sea	-	-	2003
	ANGBF9524-12	Turkey, Medit. Sea Or.	-	-	2003
	ANGBF9527-12	France, Medit. Sea occ.	-	-	2003
	ANGBF9528-12	France, Medit. Sea occ.	-	-	2003
	ANGBF9529-12	France, Medit. Sea occ.	-	-	2003
	ANGBF9757-12	Italy, Medit. Sea central	-	-	2003
	SDP95005-13	USA, California	34.0633	-119.818	2013
	SDP95009-13	USA, California	34.0633	-119.818	2013
	SDP125013-14	USA, California	34.063	-119.818	2013
	FISHP156-15	Peru. Pacific Ocean	-12.129	-77.234	-
***Scomber colias***	CSFO152-10	Italy, Sicily	-	-	-
	CSFO153-10	Italy, Sicily	-	-	-
	CSFO154-10	Italy, Sicily	-	-	-
	DSFSF201-09	SouthAfrica, B. Agulhas	-	-	2008
	DSFSF202-09	SouthAfrica, B. Agulhas	-	-	2008
	FCFPS032-06	Portugal, Faro, Algarve	Sub-area IXa		-
	FCFPS033-06	Portugal, Faro, Algarve	Sub-area IXa		-
	FCFPSW057-06	Portugal	40.12	-9.07	2005
	FCFPSW059-06	Portugal	40.12	-9.07	2005
	FCFPSW119-06	Palestine	40.57	-9.29	2005
	JFS461-14	Palestine	-	-	2014
	JFS462-14	Palestine	-	-	2014
	JFS463-14	Palestine	-	-	2014
	JFS464-14	Palestine	-	-	2014
	JFS465-14	Palestine	-	-	2014
	JFS466-14	Palestine	-	-	2014
	MLFPI 234-11	South Portugal, At. Ocean	37.05	-8.75	2011
	TZMSA018-04	East SouthAfrica	-30.333	30.75	2003
	TZMSA020-04	East SouthAfrica	-30.333	30.75	2003
***Scomber australasicus***	ABFJ 065-06	Japan, Pacific Ocean	35	139.5	-
	ABFJ 066-06	Japan, Pacific Ocean	35	139.5	-
	FSCS 554-07	China, China Meridional Sea	19.685	112.767	2007
	FSCS 555-07	China, China Meridional Sea	19.685	112.767	2007
	FSCS 556-07	China, China Meridional Sea	19.685	112.767	2007
***Scomber scombrus***	ANGBF 9519-12	France, East Medit. Sea	-	-	2003
	ANGBF 9521-12	Greece, East Medit. Sea	-	-	2003
	ANGBF 9753-12	Germany, North Sea	-	-	2003
	ANGBF 9754-12	Germany, North Sea	-	-	2003
	CSFOM 066-10	Italy, Sicily	-	-	-
	DNATR 1369-13	Turkey, Aegean Sea	-	-	-
	DNATR 1370-13	Turkey, Aegean Sea	-	-	-
	DNATR 1371-13	Turkey, Aegean Sea	-	-	-
	FCFP 097-05	Portugal, Atlantic ocean	38.58	-9.43	2005
	FCFP 098-05	Portugal, Atlantic ocean	38.58	-9.43	2005
	FCFP 099-05	Portugal, Atlantic ocean	38.58	-9.43	2005
	FCFP S152-06	Portugal, Faro, Algarve	Sub-area IXa		-
	FCFP W017-06	Portugal	40.02	-9.08	2005
	FCFP W029-06	Portugal	39.97	-9.37	2005
	FCFUK 041-06	Great Britain, Liverpool	53.23	-3.37	2006
	FCSF 178-14	France	Depart. code 86		2013
	FCSF 202-14	France	Depart. code 75		2013
	FOA 800-04	Great Britain, Plymouth	-	-	2001
	SCFAC 454-06	Canada, Nova Scotia	43.018	-62.142	-
	SCFAC 466-06	Canada, Nova Scotia	43.018	-62.142	-
	SCFAC 264-06	Canada, Nova Scotia	41.425	-66.301	-
	SCFAC 837-06	Canada, G.St. Lawrence	45.771	-61.858	2006
	SCFAD 497-09	Canada, Nova Scotia	44.022	-59.014	2007
***Sarda sarda***	MLFPI209-11	Sesimbra, Portugal	38.44	-9.1	2011
	MLFPI210-11	Sesimbra, Portugal	38.44	-9.1	2011
	MLFPI220-11	Sesimbra, Portugal	-	-	2011

## RESULTS AND DISCUSSION

Analysis of the 73 COI gene sequences revealed 94 variable sites in the genus *Scomber* from different regions, including those corresponding to Argentine mackerels. These decreased to 34 when it was made between *S*. *japonicus* and *S. colias* and 26 if only specimens of *S. japonicus* were considered. Within this latter group 10 variable sites were found in *S. japonicus* (excluding those obtained from the Pacific Ocean and Argentina), 14 corresponding to *S.* *japonicus *from the Pacific and one belonging to Argentina. Most of these variable sites were transitions (CT or AG). Similar results were found in a study by Cheng et al. [19] with COI, cyt b and control region sequences who described an excess of transitions with respect to transversions in mitochondrial genes.

Average estimate of nucleotide diversity (π) considering all *Scomber* species was 0.05 ± 0.01. Haplotype diversity of *S. japonicus* was high (Hd = 0.71 ± 0.073) and nucleotide diversity low (π = 0.008 ± 0.0016). The average nucleotide diversity was 0.001 ± 0.001 for Argentine mackerels; between Argentina and the rest of *S. japonicus* was 0.011 ± 0.003, while between Argentina and *S. colias* or *S. japonicus* (not Pacific) was similar, 0.003 ± 0.001 and 0.005 ± 0.002, respectively.

The distances based on K2-P model between species of the same genus resulted in a mean of 0.064. Distance values, measured as the number of base substitutions per site, from averaging all pairs of sequences between the groups, are shown in Table 2. The analysis included 76 nucleotide sequences of *Scomber* genus and *Sarda sarda*, resulting in a total of 310 positions in the final data set. The values obtained were similar to those found in previous studies: 0.015 between *S. japonicus* and *S. australasicus* and 0.16 between both and *S. scombrus* [17]; 0.019 between *S. japonicus* and *S. australasicus* and 0.14 between these two and *S. scombrus* [31]; 0.017 and 0.019 between *S. japonicus* - *S. australasicus* and *S. japonicus* - *S. colias*, respectively, and 0.2 between any of these three species and *S. scombrus* [19]. The results presented in Table 2 are consistent with all previous studies, where it is also noted that *S. scombrus* is the most divergent species of the genus *Scomber*.

**Table 2:** Evolutionary divergence estimates between pairs of sequences between groups

***S. japonicus_ARG***	0						
***S. japonicus***	0.003	0					
***S. japonicus_ PAC***	0.019	0.017	0				
***S. colias***	0.001	0.005	0.02	0			
***S. scombrus***	0.113	0.112	0.120	0.114	0		
***S. australasicus***	0.016	0.018	0.028	0.017	0.113	0	
***Sarda sarda***	0.206	0.205	0.209	0.207	0.175	0.192	0

On the other hand, distances between *S. japonicus* and *S. colias* were low, consistent with those found by Catanese et al. [9], who concluded that *S. japonicus*, *S. colias* and *S. australasicus* are very closely related species. They even considered that the values found are more appropriate for populations belonging to the same species (for COI, between 0.016 and 0.024) than species of the same genus. These authors speculated that this closeness could be due to past gene flow and hybridization by introgression after speciation. A 3% threshold has been postulated as the level of genetic variation that characterizes different animal species [32]. However, these values are lower among fish and it has been indicated that at divergence ranges greater than 2%, probability of conspecificity is very low for fish species, whereas in the range of 2% to 4% congeneric comparisons predominate [33]. Distance values obtained for *Sarda sarda* regarding *Scomber* are in accordance with the latter mention.

Intraspecific distances were low, except in *S. japonicus* in which a value of 0.01 ± 0.003 was obtained due to sequences of members from different regions, including Argentina. By disaggregating these representatives, the values obtained were lower (Table 3). The highest value found for Pacific *S. japonicus* is due to the presence of Eastern and Western Pacific representatives. Similar values were found in other fish studies: species of salmon and trout in North America, 0.27% [34]; 194 species from Canada fish fauna 0.30% (0 to 7.42%) [35]; and Ward et al. [36] recorded 0.39% (0-14.08%) in 207 marine species in Australia. Also, generally 95% of within species values are less than 2% [37].

**Table 3:** Average distance values between pairs of sequences within each group and standard deviation (SD

	D	SD
*S. japonicus_ARG*	0	0
*S. japonicus*	0.006	0.008
*S. japonicus_PAC*	0.012	0.008
*S. colias*	0.003	0.004
*S. scombrus*	0.001	0.004
*S. australasicus*	0.005	0.006
*Sarda sarda*	0.020	0.01

**Figure 1:**
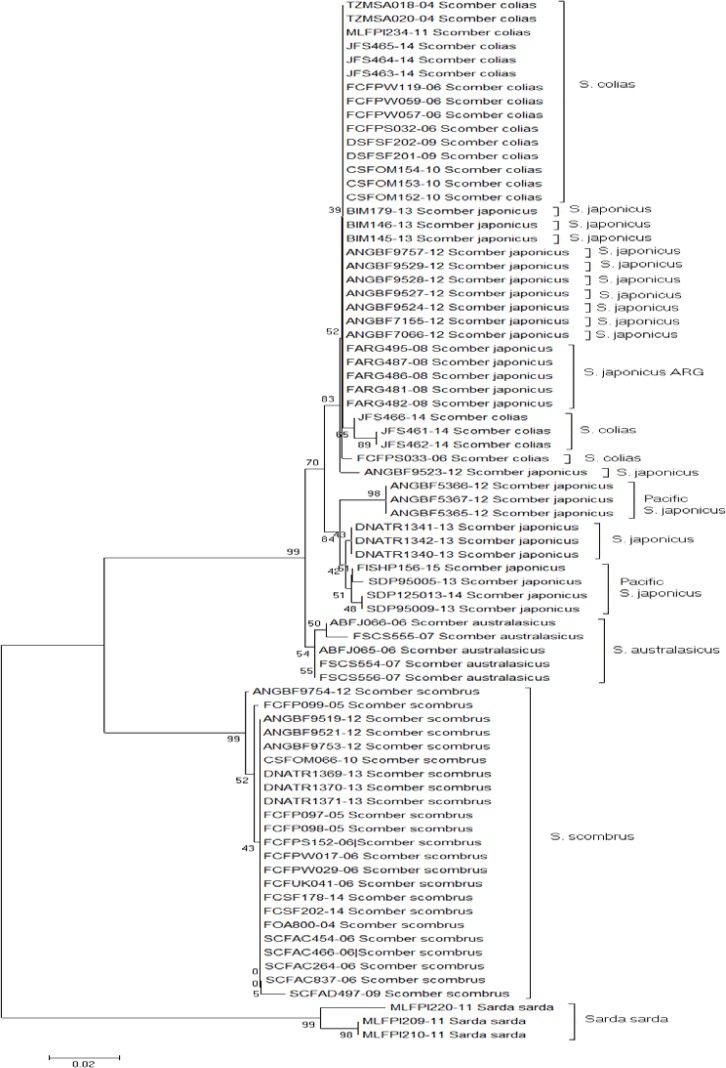
Neighbor joining tree topology from the K2P model of 76 sequences corresponding to *Scomber* and *Sarda sarda*. Accession number sequences as in Table 1

**Figure 2:**
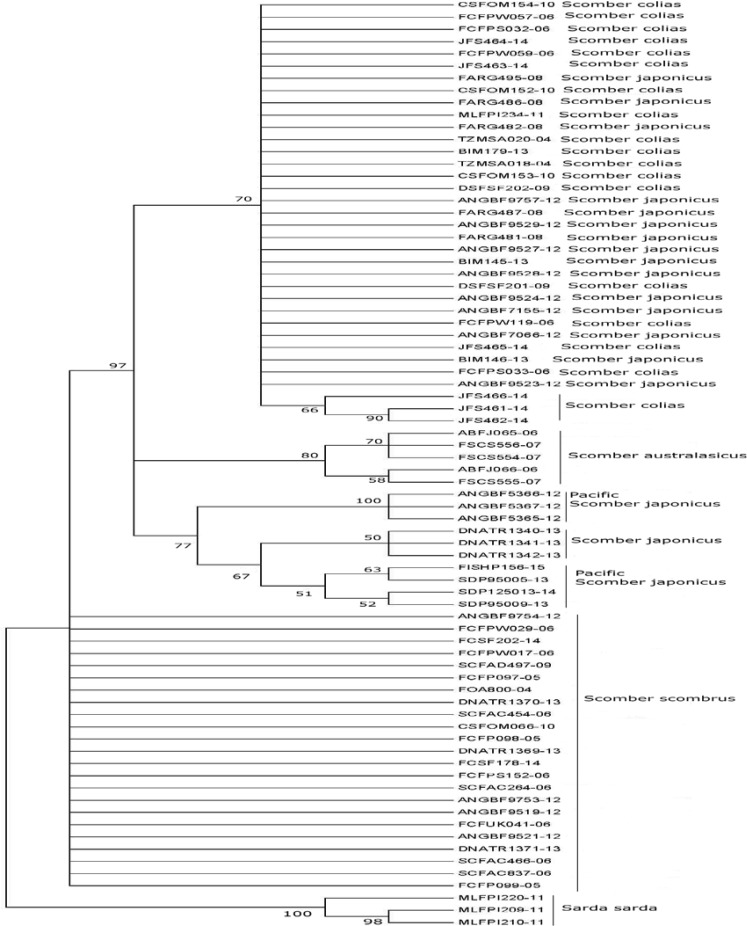
Tree topology based on maximum likelihood method and evolutionary model GTR + G. Accession number as in Table 1

The tree generated by Neighbor joining based on COI sequences showed that most of the specimens were clustered according to their taxonomic classification: first the rooting of the tree considering *Sarda sarda* as outgroup was observed, and second, nearby genetic species were clustered under the same node (*S. colias* and *S. japonicus*), with a 70% bootstrap value. In turn, this set was grouped with *S. australasicus* with a reliability of 99%. However, the results for Argentine mackerels (FARG-) did not reflect the current taxonomic classification. Same deviation was observed in mackerels of Palestine (BIM 179-13 145,146), Italy (ANGBF9757-12), France (ANGBF9527 to 9529-12), Turkey (ANGBF9524-12) and South Africa (ANGBF7066 and 7155-12). The bootstrap value of this node was less than 50% (Fig. 1). Although Argentine Sea mackerel as well as those just mentioned are listed in the FISH-BOL database as *S. japonicus*, all clustered with *S. colias*. This can be explained from distance values obtained between sequences of both species (Table 2). The maximum likelihood tree showed a similar topology: Argentine sequences as well as those belonging to *S. japonicus* mentioned previously (Israel-Italy-France-Turkey-South Africa), were grouped with *S. colias*, with a higher bootstrap value, 70% (Fig. 2). Earlier studies have noted differences in *S. colias* position as a sister group of *S. japonicus* or *S. australasicus*, depending on the molecular marker used [8, 9]. According to Cheng et al. [19] this could be due to the sequences length of each marker, so using longer sequences was advised to get a higher resolution in conflict species.

From these results, haplotypes were determined from 45 sequences including Argentine mackerels (FARG-), those corresponding to *S. japonicus* (including the Pacific) and *S. colias*, resulting in 14 different haplotypes, excluding gaps and missing data. In Argentine mackerels two haplotypes, H1 and H2, were recognized. The H1 haplotype was the most frequent (53%), and was shared by four of the five Argentine sequences, seven sequences of *S. japonicus* (South Africa, Turkey, France, Italy and Israel, agreeing with the tree topology) and 13 sequences *S. colias* from South Africa, Italy, Palestine and Portugal. Haplotype 2 was unique and corresponded to the sequence of the third FARG-sequence (Table 1). 

**Figure 3:**
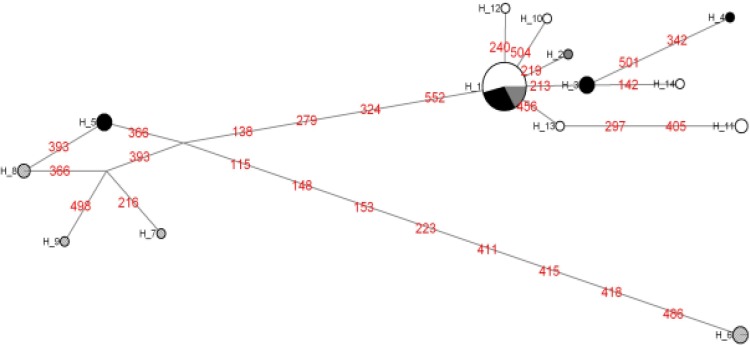
*S. japonicus* and *S. colias* network haplotypes from 45 sequences of mitochondrial COI gene. The circles represent haplotypes (14) and areas are proportional to the number of sequences in both species. Numbers in lines represent mutations in the nucleotide site. White areas represent *S. colias*, black *S. japonicus*, striped Pacific S*. japonicus* and solid gray to Argentine mackerels

The identified haplotypes are shown in Table 4 where the first three lines details the position of the variable site. In the haplotypes network generated by median joining, the relationship between the different sequences of *S. japonicus* and *S. colias* (Fig. 3) was observed. Between haplotype 1 and 2, corresponding to Argentine sequences, there was a difference of a mutational step and a closer observation of the sequences indicated a transition from C to T. In addition, the proximity of the remaining haplotypes of *S. colias* (10, 12, 13, 14 and 11) and *S. japonicus* (3 and 4) belonging to South Africa, France (Western Mediterranean) and Spain (Cantabria) samples was observed, with one or two mutational steps among them. On the other hand, haplotypes of Eastern Pacific *S. japonicus* (7, 8 and 9 from the USA and Peru) and haplotype 5, belonging to sequences from Turkey, were pooled together, with more than four mutational steps regarding the most frequent haplotype. Representatives of Western Pacific* S. japonicus*, from the China Sea corresponds to haplotype 6. The combined analysis of phylogenetic tree and haplotype network showed high genetic similarity between Argentine mackerel sequences and *S. colias*.

**Table 4:** Haplotypes identified from 26 variable sites corresponding to 45 sequences of *S. japonicus*

Hap_1	11111222222233334444444555
	13445111247924690111589005
	58283369309742635158668142
	ATGGAAGCGTAGTTGAACGGAAACTT
Hap_2	.......T..................
Hap_3	.....G....................
Hap_4	.....G.......C.........T..
Hap_5	.C........C.C.A..........C
Hap_6	TC.AG...C.C.C....GAA.G...C
Hap_7	.C....A...C.C..G.........C
Hap_8	.C........C.C.AG.........C
Hap_9	.C........C.C..G......G..C
Hap_10	........................C.
Hap_11	...........A....G...C.....
Hap_12	.........C................
Hap_13	....................C.....
Hap_14	..T..G....................

The results of this study showed that:

- The analysis of COI gene sequences allowed differentiation of the four species of *Scomber* accepted as valid in the current taxonomy. The sequences obtained from Argentine mackerels were associated with *S. colias*, with genetic differences corresponding to conspecific populations (0.1%).
- Neighbor joining and maximum likelihood trees based on distances between pairs of sequences or characters, respectively, provided evidence of a classification of the Argentinean mackerel that does not match the current taxonomic position.
- The haplotype network generated by median joining showed that four of the five Argentine specimens shared the same haplotype with *S. colias*, and none was shared with *S. japonicus* from Pacific Ocean.
- The high genetic similarity found in this study supports the proposal of renaming Argentine mackerels to Atlantic chub mackerel, *S. colias*.
